# A simulation-based dataset for anomaly detection in hydrogen blend transport networks

**DOI:** 10.1016/j.dib.2026.112520

**Published:** 2026-01-28

**Authors:** Andrea Senese, Saverio De Vito, Elena Esposito, Michele Villari, Giovanni Acampora, Girolamo Di Francia, Antonia Longobardi, Giulia Monteleone

**Affiliations:** a Department of Physics "Ettore Pancini", University of Naples Federico II, Via Cinthia,21 (Building 6), Naples 80126, Italy; b TERIN-SSI-EDS Laboratory - ENEA CR-Portici, Piazzale Enrico Fermi, 1, Portici 80055, Italy; c ENEA, Lungotevere G.A. Thaon di Revel, 76, Rome 00196, Italy; d Department of Civil Engineering, University of Salerno, Via Giovanni Paolo II, Fisciano 84084, Italy

**Keywords:** Hydrogen network modeling, Hydrogen network diagnostics, IoT sensors, Anomaly detection, Clean energy, Hydrogen natural-gas blending

## Abstract

Hydrogen transport involves the safe movement of gaseous hydrogen through industrial pipeline networks, typically between production plants, storage facilities, and distribution centers, and is a key component in the transition toward more sustainable energy sources [1]. Monitoring these networks is essential, as hydrogen is highly flammable and leaks, compressor failures, or delayed component responses can lead to serious accidents, environmental damage, and operational interruptions. Despite the growing interest in this sector, publicly available datasets containing multivariate data on hydrogen transport networks are extremely limited, hindering the development and evaluation of data-driven monitoring methods [[2], [3], [4]]. To address this gap, we present a synthetic dataset simulated using a MATLAB Simscape model of a pipeline segment representative of an industrial network [[5], [6], [7],^14^]. The dataset includes time-series data from distributed virtual sensors, covering both normal operating conditions and anomalous scenarios such as leaks, compressor failures, and delayed component responses [8,9]. The simulation reproduces transient and steady-state dynamics typical of industrial networks, providing data suitable for the development and evaluation of algorithms for digital twins [10], monitoring, and anomaly detection in hydrogen transport infrastructures [10,11].

Specifications Table

**Table:** 

Subject	Computer Sciences
Specific subject area	Hydrogen transport networks, focusing on monitoring, digital twins, and anomaly detection using time-series data and simulation-based methods.
Type of data	Multivariate Time-series Data.
Data collection	Data were generated using a MATLAB Simscape model simulating a hydrogen pipeline segment. The dataset includes 12 distributed virtual pressure sensors and 4 mass flow rate sensors, capturing both normal operating conditions and anomalous scenarios such as leaks, compressor failures, and delayed component responses. The simulation reproduces both transient and steady-state dynamics typical of industrial hydrogen transport networks.
Data source location	University of Naples Federico II and ENEA Research Center.
Data accessibility	Repository name: Zenodo Data identification number: 10.5281/zenodo.17871393 Direct URL to data: https://doi.org/10.5281/zenodo.17871393
Related research article	None.

## 1 Value of the Data

• The dataset contains multivariate time series simulated with a MATLAB/Simscape model of a hydrogen transport network, including both normal operating conditions and engineered anomalies such as leaks, compressor faults, and component startup delays.
• The dataset is intended for researchers and practitioners working on anomaly detection, fault/leak diagnosis, and condition monitoring in energy transport infrastructures, with particular relevance for hydrogen-blend pipeline networks. It supports research activities such as algorithm development, validation, and benchmarking, as well as simulation-based studies relevant to industrial monitoring and decision-support applications where access to real operational data is limited.
• The simulation reproduces transient and steady-state dynamics typical of industrial infrastructures, with added Gaussian white noise to represent sensor uncertainties. This combination of transient and multivariate sensor signals provides a detailed view of the network’s dynamic behavior, allowing anomaly detection algorithms to distinguish between normal operational fluctuations and potentially anomalous events, even in the presence of overlapping or faults [5,[18], [19]].
• The dataset represents a unique resource for the scientific community, as it captures hydrogen-specific transient behaviors and multivariate sensor data along the network, covering both normal and anomalous scenarios. Compared to existing resources, such as GasLib [15], commercial tools like SimGas or InfoWorks WS Pro Gas [16], and open-source simulators such as DWSIM [17], this dataset allows for the study of realistic anomaly detection problems where small transient changes in sensor signals indicate early-stage faults.
• The three operational topologies considered (gest_down, gest_center, gest_up) differ only in the location of the active withdrawal point along the network. These points were chosen to provide three distinct withdrawal scenarios along the same network segment, inspired by, but not intended to replicate, real industrial hydrogen transport infrastructure (e.g., SNAM [13]). Activating multiple branches simultaneously would create numerical conflicts in the differential equations governing the transient simulation, potentially causing solver instability. Limiting the activation to a single branch ensures numerical stability, preserves physical fidelity, and still generates structural variability in the data across different configurations, effectively providing a form of “data augmentation” for testing anomaly detection robustness. This approach increases the number of topologies and the diversity of operational conditions in the dataset, allowing anomaly detection algorithms to be tested more thoroughly against realistic variations.
• The data are structured and labeled to support anomaly detection, classification, and localization, as well as studies on sensor placement optimization.
• It can be used to develop, test, and benchmark monitoring, digital twin [12], and robust anomaly detection algorithms in simulations representative of real-world conditions.

## 2 Background

The simulation of the hydrogen transport network is based on thermo-fluid dynamic models that describe the evolution of key quantities such as pressure, density, velocity, and mass flow along the pipelines. The fundamental laws considered include conservation of mass, momentum, and energy, coupled with the real gas equation of state. The model reproduces both steady-state and transient dynamics typical of industrial networks, taking into account phenomena such as internal friction, heat exchange with the environment, and pipeline slope. This simplified approach allows the generation of realistic time-series data suitable for testing algorithms for monitoring, digital twins, and anomaly detection, without needing to solve every mathematical detail of high-pressure compressible gas flows.

## 3 Data Description

### 3.1 Simulation model of the hydrogen transport network

The hydrogen transport network was modeled in MATLAB/Simscape (R2025a) using components from the Simscape Gas Fluids library.

The network includes:

• Pressurized supply tank providing hydrogen at constant initial pressure and temperature.
• Pipelines and nodes representing conduits and junctions.
• Local restrictions (G1–G6) and compressors (C1–C3) to simulate operational components and inject controlled anomalies.
• Constant Volume Chamber (V) to model confined gas volumes.

Twelve pressure sensors (PS1–PS12) and four mass flow sensors (MFS1–MFS4) are placed at strategic locations to capture system dynamics. Three normal operating topologies were simulated, each corresponding to a different withdrawal point (downstream, central, upstream). Each scenario spans approximately 20,000 s (∼5.5 hours) and reproduces the four operational phases: initial ramp-up, transient, steady-state, and shutdown.

The simulated model includes pressurized supply tanks, compressors, pipelines with varying geometrical characteristics, constant volume chambers, and local restrictions. Key physical parameters for the **gest_down** topology are as follows: the controlled tank has a cross-sectional area of 1.1340 m² at port A; compressor G3 has cross-sectional areas of 1.1340 m² at ports A and B, operates as a controlled source, and injects power isentropically. Pipelines have lengths between 3000 m and 30,000 m, hydraulic diameters between 0.4 m and 1.2 m, and corresponding cross-sectional areas between 0.1256 m² and 1.1304 m². Local restrictions are fixed, with an area of 1e^−3 m², discharge coefficient 0.64, and laminar flow pressure ratio 0.999. Constant volume chambers (1 m³) are positioned in branches with sensors to simulate gas accumulation and dead volume dynamics, allowing correct simulation of pressure and flow variations during leaks or valve closures. Other compressors and tanks are configured with specific area, pressure, and power values as directly set in the Simscape model. These parameters were selected to be internally consistent and plausible for a hydrogen transport network, allowing reproducible simulations and coherent data generation for anomaly detection and digital twin studies, while not claiming to fully represent all features of real industrial networks.

### 3.2 Dataset composition

The dataset provides multivariate time series with synchronized measurements from all sensors.

Each record corresponds to a single time step (sampling interval ≈ 1 s) and includes the following fields and multi-label anomaly indicators (Table 1 and 2).

**Table 1:** Fields and multi-label anomaly indicators included in each record of the dataset.

Field / Label	Type	Description
PS1–PS12	Float	Pressure measurements from 12 sensors [MPa]
MFS1–MFS4	Float	Mass flow measurements from 4 sensors [kg/s]
label_anomaly	Binary	0 = Normal, 1 = Anomaly
Leak	Binary	Leak detected (any valve)
CompressorFault	Binary	Compressor fault detected
CompressorDelay	Binary	Compressor startup delay detected
leak_G3, G4, G5, G6	Binary	Leak localized at specific valve

**Table 2:** Injected anomalies in the hydrogen transport network simulations.

Component	Anomaly Type	Intensity	Start Time	Operational Phase
G3	Leak	Strong	4000 s	Steady State
G4	Leak	Weak / Strong	2500 s	Steady State
G5 + G6	Overlapping Leaks	Strong / Weak	400 s	Late Transient
C1	Compressor Fault	Weak / Strong	25 s	Initial Ramp-up
C1	Compressor Start Delay	Strong	1000 s	Initial Ramp-up

### 3.3 Fields included in each record

Time series are generated for multiple operational topologies and scenarios, both under normal conditions and with injected anomalies. Each scenario provides a continuous sequence of ∼20,000 records (∼5.5 hours) covering all operational phases. This structure supports anomaly detection, classification, and localization studies.

Pressure sensors are placed before and after pipelines, compressors, and local restrictions to monitor pressure profiles along the network and detect significant variations caused by anomalies. Mass flow sensors are located near valves and potential leak points to detect flow changes due to faults or leaks. Sensor numbering and placement are consistent across the three operating topologies (gest_down, gest_center, gest_up), ensuring a fixed feature space for machine learning models even when a branch is inactive. This layout maximizes informative data while reducing non-significant signals.

### 3.4 Anomaly injection

Anomalies were introduced in the simulation using different components.

Multiple anomalies may occur simultaneously.

### 3.5 Summary of injected anomalies

Sensor Configuration and Noise Injection

To replicate realistic industrial sensor behavior, Gaussian white noise was added to all signals:

noisy_signal=signal+σ·randn(N)

where σ corresponds to a moderate SNR (≈1–2 %). The chosen SNR level is based on typical specifications of industrial sensors reported in the literature [11, Table 3, p. 272], which list the noise characteristics of pressure, flow, and temperature sensors commonly used in hydrogen transport networks and other energy-related industrial processes. This moderate SNR reflects realistic sensor performance in operational environments and ensures that the dataset accurately represents measurement uncertainties without introducing arbitrary noise. For transparency and reproducibility, the Zenodo repository associated with the dataset also includes the file used to inject noise into the measurements, allowing readers to verify the implementation of realistic sensor noise characteristics.

**Table 3:** Organization of the H2-SimNet dataset files.

Folder	Contents
anomalous_scenarios/	Scenarios with anomalies and Gaussian noise
H2-SimNet-clean/	Version of the dataset without noise
normality_scenarios/	Normal scenarios without anomalies
MATLAB_SIMSCAPE_Simulation/	Original MATLAB Simscape files

### 3.6 Dataset files and structure

The dataset is organized to support reproducible anomaly detection experiments. Data are provided in **CSV** and **Parquet** formats, with separate folders for normal and anomalous scenarios.

A metadata PDF (README) includes:

• Network topology and sensor placement
• Scenario configurations (anomaly types, timings, intensities)
• Mapping of simulation outputs to sensor and label fields

**Dataset Organization**

## 4 Experimental Design, Materials and Methods

### 4.1 Dataset files and formats

The dataset is organized to facilitate reproducible experiments in anomaly detection and monitoring. All acquired data are provided in two formats, each stored in a separate folder:

• **CSV/** – contains the data acquisition in CSV format
• **Parquet/** – contains the data acquisition in Parquet format

Each format includes subfolders for each topology, with further subdivisions for **normal** and **anomalous** scenarios.

The CSV format ensures maximum compatibility with general-purpose software and can be read without programming environments, while the Parquet format, being columnar and binary, reduces reading times and memory usage and allows selective access to only the columns of interest, making it more efficient for automated analyses and Python-based workflows.

The data acquisition files include:

• 12 pressure sensors (PS1–PS12)
• 4 mass flow sensors (MFS1–MFS4)
• Local restrictions (G1–G6) and compressors (C1–C3), which are also used to generate controlled anomalies

The hydrogen transport network was modeled in MATLAB Simscape, initially developed in **R2024b** using Simscape Fluids. The model was later opened in **R2025a** to verify possible improvements or newly available components, without observing significant differences in functionality or network modeling. The model files can also be opened in earlier compatible MATLAB/Simscape versions (e.g., R2024a/b) and can be exported in a backward-compatible format. Researchers encountering difficulties with older versions can request assistance or adapted model files to facilitate reproducibility.

**Fig. 1:**
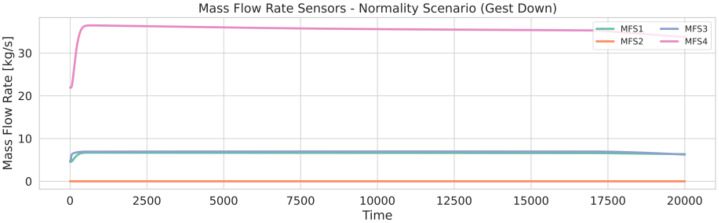
Illustration of the time-series visualization of the pressure sensor measurements (PS1–PS12) recorded during a normal operating scenario of the hydrogen transport network.

**Fig. 2:**
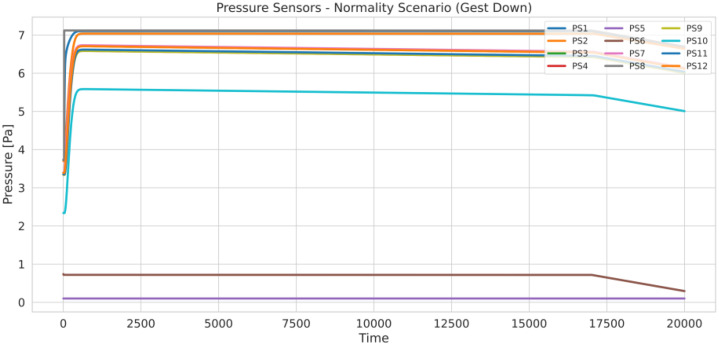
Illustration of the time-series visualization of the mass flow rate sensor measurements (MFS1–MFS4) recorded during a normal operating scenario of the hydrogen transport network.

After detailing the structure and content of the dataset files, Fig. 1, Fig. 2 illustrate example time-series plots from the dataset under normal operating conditions. In these figures, the structure and behavior of the signals reflect the operational dynamics of the hydrogen transport network, which can be divided into four main phases:

1. **Initial ramp-up**
2. **Transient**
3. **Steady state**
4. **Closing phase**

Each phase exhibits characteristic pressure and flow patterns determined by the network model, sensor placement, and simulated control actions. In the closing phase, visible toward the end of the plots, the pressure begins to decrease just before 20,000 s, simulating the gradual shutdown of the network.Understanding these phases is essential for interpreting how anomalies such as leaks, compressor faults, or valve restrictions manifest in the sensor measurements data.

The dataset can be used within a digital twin workflow for hydrogen transport networks. In particular, the simulated multivariate data are extracted from the simulation, organized into multivariate time-series, and used to train and validate anomaly detection models. **In a typical workflow, these data feed the digital twin, the models process the time-series, and provide a supervisory and diagnostic layer for the network, comparing the observed behavior with the nominal simulated conditions.** In this way, the dataset serves as a controlled reference for the calibration, testing, and validation of digital twin monitoring systems before integration with real operational data.

## Ethics Statement

The authors declare that this work does not involve the use of human subjects or experimentation with animals.

## CRediT Author Statement

**Giovanni Acampora:** Supervision, Review; Saverio De Vito: Supervision, Review; **Elena Esposito:** Supervision, Review; **Giulia Monteleone:** Review; **Antonia Longobardi:** Supervision, Review; **Michele Villari:** Conceptualization, Simulation Implementation; **Andrea Senese:** Conceptualization, Simulation Extensions, Methodology, Software, Implementation, Investigation, Data Curation, Writing - original draft, Corresponding Author.

## Data Availability

ZenodoH2-SimNet Dataset: A Simulation-based Dataset for Anomaly Detection in Hydrogen Blend Transport Networks (Original data)

## References

[1] (2023). Hydrogen Transport and Storage Networks Pathway.

[2] Gerard B., Carrera E., Bernard O., Lun D. (2022). Smart design of green hydrogen facilities: a digital twin-driven approach. E3S Web Conf..

[3] T. Brunner and O. Kircher, “Cryo-compressed hydrogen storage,” 2016. 10.1002/9783527674268.ch29

[4] Xie Z., Jin Q., Su G., Lu W. (2024). A review of hydrogen storage and transportation: progresses and challenges. Energies.

[5] González Herrán, Cruz Alberto de la, Jesús Andrés-Toro B., Risco-Martín J.L. (2009). Modeling and simulation of a gas distribution pipeline network. Appl. Math. Model..

[6] Saif R. Kazi, Kaarthik Sundar, Anatoly Zlotnik. “Dynamic optimization and optimal control of hydrogen blending operations in natural gas networks,” 2024, 2304.02716, arXiv. Available: https://arxiv.org/abs/2304.02716

[7] Villari M., Longobardi A., De Vito S. (2024). Master’s thesis in Civil Engineering.

[8] A. Jaribion, S.H. Khajavi, M. Öhman, A. Knapen, J. Holmström, (2020). A digital twin for safety and risk management: a prototype for a hydrogen high-pressure vessel. In: Hofmann, S., Müller, O., Rossi, M. (eds) *Designing For Digital Transformation. Co-Creating Services With Citizens and Industry. DESRIST 2020. Lecture Notes in Computer Science*, vol 12388. Springer, Cham. Available: 10.1007/978-3-030-64823-7_34

[9] El-Amin M.F. (2024). 21st Learning and Technology Conference (L and T).

[10] Ghosh R., Kesarwani S., Malhotra M. (2025). A mini review on smart hydrogen: the role of AI in revolutionizing green hydrogen systems. Energy Fuels.

[11] K. Shaheen, A. Chawla, F.E. Uilhoorn and P.S. Rossi, "Sensor-fault detection, isolation and accommodation for natural-gas pipelines under transient flow," in *IEEE Transactions on Signal and Information Processing Over Networks*, vol. 10, pp. 264–276, 2024, 10.1109/TSIPN.2024.3377134. Available: https://ieeexplore.ieee.org/document/10472095

[12] Feng Z., Luo Y., Li D., Pan J., Tan R., Chen Y. (2025). Integrating digital twins and machine learning for advanced control in green hydrogen production. Chain.

[13] SNAM, “Gas network and gas transport infrastructures,” [Online]. 2025. Available: https://www.snam.it/it/i-nostri-business/trasporto/informazioni-commerciali/rete-gas-e-infrastrutture-trasporto-gas.html

[14] Villari M., De Vito S., Esposito E., Senese A., Longobardi A., Di Francia G., Di Natale C., Lorenzelli L., Mulloni V. (2025). Sensors and Microsystems. AISEM 2025, Lecture Notes in Electrical Engineering.

[15] D. De Wolf, Y. Smeers, *GasLib — A Library of Gas Network Instances*, **Data**, vol. 2, no. 4, p. 40, 2020. Available: https://www.mdpi.com/2306-5729/2/4/40.

[16] Liana V., Piraianu V. (2008). Proceedings.

[17] Rosa C., Satyro M., Svrcek C., Young W., Brent B. (2003). Proceedings of the IASTED International Conference on Modelling and Simulation.

[18] Shaheen K., Chawla A., Uilhoorn F.E., Rossi P.S. (2024). https://ieeexplore.ieee.org/document/10614236.

[19] Ma J., He Y., Su H., Zhang J. (2024). 2nd International Conference on Artificial Intelligence and Automation Control (AIAC).

